# Health Primers

**Published:** 1879-05

**Authors:** 


Health Primers; D. Appleton & Co., N. Y.

These Primers constitute a series of four separate monographs, neatly bound in cloth and treating of the following subjects:

No 1. Exercise and Training; By C. H. Ralfe, M. D.

No. 2. Alcohol, Its Use and Abuse; By W. S. Greenfield, M. D.

No. 3. The House and Its Surroundings.

No. 4. Primitive Death, Its Promotion and Prevention.

This series is to be extended and the following corps of editors and contributors serve to reccommend the enterprise, viz:

Editors—J. Langdon Down, M.D., etc.; Henry Powers, M.B., etc.; J. Mortimer Granville, M.D.; John Tweedy, F.R.C.S.

Contributors to the series—G. W. Balfour, M.D., etc.; J. Crichton Browne, M.D.; C. W. Heaton, F.C.S., etc.; and many others whose names are guarantees that the series will possess much of a scientific and beneficial character.

